# Direction of pelvic obliquity after total hip arthroplasty for dysplastic hip osteoarthritis: a retrospective observational study

**DOI:** 10.1007/s00402-025-05829-5

**Published:** 2025-03-19

**Authors:** Hiroyuki Yokoi, Yusuke Osawa, Yasuhiko Takegami, Yuto Ozawa, Hiroto Funahashi, Shiro Imagama

**Affiliations:** Nagoya University Hospital, Nagoya, Japan

**Keywords:** Pelvic obliquity, Total hip arthroplasty, Dysplastic hip osteoarthritis, Clinical outcomes

## Abstract

**Introduction:**

Pelvic obliquity (PO) in dysplastic hip osteoarthritis (DHOA) can present as either upward or downward tilting of the affected side. This study investigated the influence of preoperative PO direction on postoperative clinical outcomes and hip–spine morphology in patients undergoing total hip arthroplasty (THA).

**Materials and methods:**

Data from 116 (21 men, 95 women) patients with unilateral DHOA, who underwent THA at a single institution between June 2018 and September 2023 and exhibited ≥ 2° of PO, were analyzed. Patients were categorized into two groups: upward PO (U-PO [≥ 2° upward tilt, *n* = 35]); and downward PO (D-PO [≥ 2° downward tilt, *n* = 81]). Patient demographic information, surgery-related factors, hip function scores, and radiographic parameters of the hip, lower limbs, and spine were compared between the groups.

**Results:**

Except for the duration of hip disorders, no significant differences were observed in patient background and surgical data between the groups. Preoperatively, the U-PO group exhibited a larger acetabular offset, greater hip adduction angle, longer functional leg length on the affected side, and greater ipsilateral convex lumbar scoliosis than the D-PO group (*P* = 0.034, *P* < 0.001, *P* < 0.001, and *P* < 0.001, respectively). Postoperatively, a greater hip adduction angle and longer functional leg length discrepancy persisted in the U-PO group compared to those in the D-PO group (*P* < 0.001 and *P* = 0.002, respectively). The median (interquartile range) residual PO was greater in the U-PO group (3° [0–4°]) than that in the D-PO group (1° [0–3°]) (*P* = 0.009). Compared with the D-PO group, the mean postoperative hip Japanese Orthopaedic Association scores were significantly lower in the U-PO group (85 [81–92] vs. 92 [85–96], *P* = 0.016).

**Conclusion:**

The U-PO group exhibited greater residual hip adduction angles, longer functional leg lengths on the affected side, and less improvement in PO after THA than the D-PO group, resulting in poorer postoperative hip function.

## Introduction

To maintain an upright posture, the human body must balance gravitational forces through the spine, pelvis, and lower limbs. In the presence of pelvic obliquity (PO), the pelvis exhibits asymmetry in the coronal plane, preventing it from achieving an ideal horizontal position [1]. Marked PO can result in improper acetabular cup placement, emphasizing its clinical importance [2]. The etiology of PO is categorized into suprapelvic, intrapelvic, and infrapelvic and is influenced by factors such as spinal deformity, hip contracture, and leg length discrepancy (LLD) [1]. Dysplastic hip osteoarthritis (DHOA) is characterized by joint deformity and restricted range of motion (ROM) and presents greater surgical challenges than primary hip osteoarthritis [3]. As DHOA progresses, hip subluxation exacerbates LLD and hip contracture, potentially leading to PO [4]. This is particularly critical because severe PO can negatively affect adjacent joints, further complicating patient and treatment outcomes.

In patients diagnosed with primary hip osteoarthritis, the pelvis typically tilts downward on the affected side. However, in DHOA, both downward and upward pelvic tilt can be observed on the affected side. The former results from limb shortening, whereas the latter is associated with compensatory mechanisms to alleviate hip pain and is attributed to factors such as weakened hip abductor muscles and hip adductor contractures [5]. Total hip arthroplasty (THA) is widely performed for DHOA, with reports of improved PO [6–8]. However, persistent postoperative PO has been linked to perceived LLD and reduced hip function [9, 10], highlighting the importance of evaluating PO, including hip–spine parameters.

In our previous study of preoperative cases, we reported that patients with upward PO (U-PO) on the affected side demonstrated poorer hip ROM than those with downward PO (D-PO). We also highlighted the associations between hip adduction angles and D-PO as well as age, subluxation percentage, and hip adduction angles with U-PO, and identified a relationship between lumbar scoliosis angles and PO direction [5]. However, changes in the direction and angle of the preoperative PO after THA and their association with postoperative hip function, morphology, LLD, and spinal alignment remain unclear.

Therefore, the objectives of the present study were as follows: to evaluate the impact of preoperative PO direction on hip–spine morphology after THA; to assess differences in PO improvement after THA based on preoperative PO direction; and to investigate whether postoperative hip function differs according to preoperative PO direction. We hypothesized that patients with U-PO on the affected side would have poorer postoperative hip function and limited PO improvement.

## Materials and methods

### Patients

**Fig. 1:**
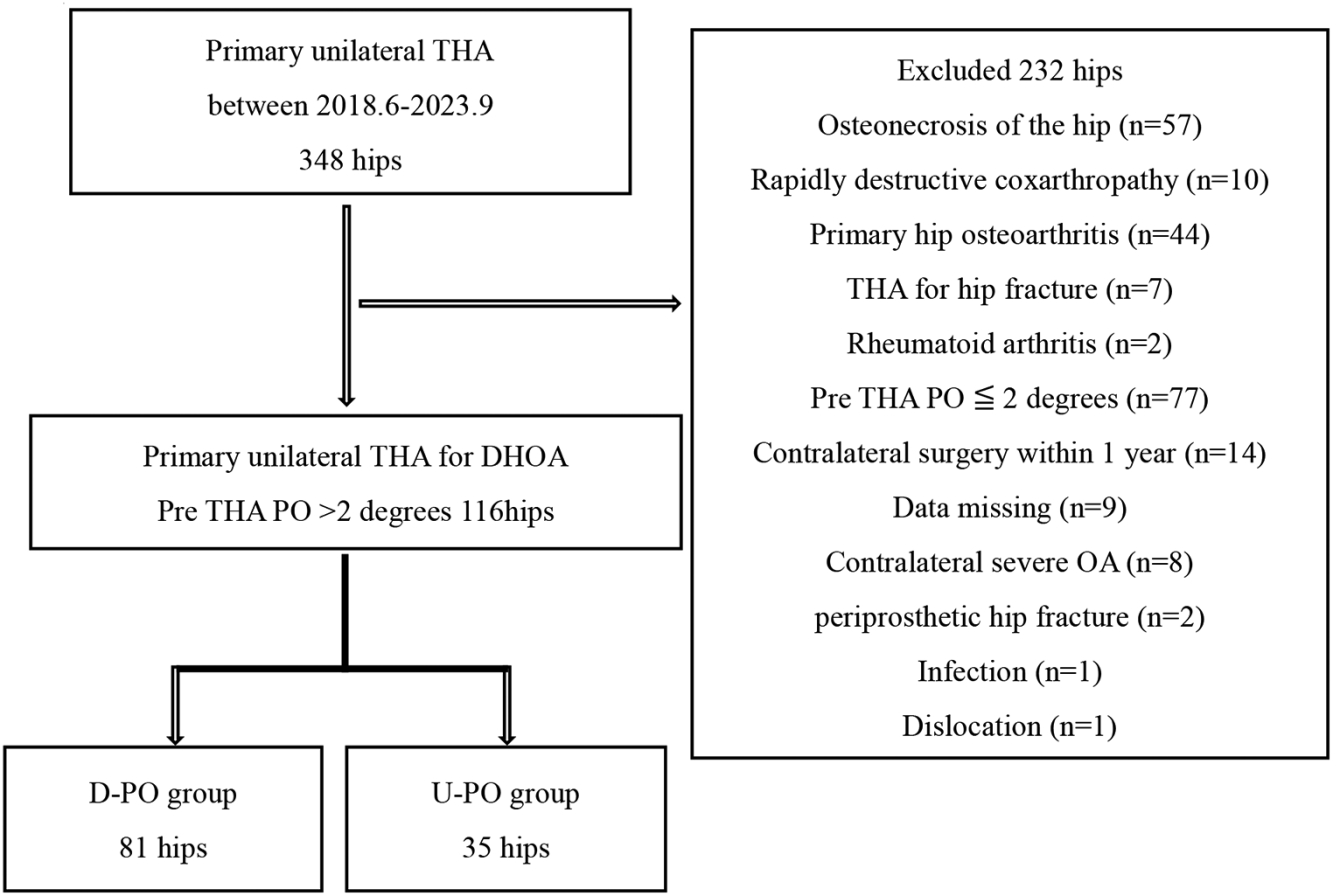
Inclusion criteria for this study. Patients with affected-side downward tilt ≥ 2° constitute the D-PO group. Patients with affected-side upward tilt ≥ 2° constitute the U-PO group

This study was approved by the Ethics Committee of Nagoya University Hospital (Nagoya, Japan). Given the retrospective observational design of this study and the use of anonymized patient data, the requirement for informed consent was waived. This retrospective study analyzed 348 patients who underwent unilateral THA at our institution between June 2018 and September 2023. From this dataset, we excluded 57 hips with femoral head necrosis, 10 with rapidly destructive coxarthropathy, 44 with primary osteoarthritis, 7 with THA for hip fracture, 2 with rheumatoid arthritis, 77 with PO < 2°, 14 that underwent contralateral surgery within one year, 9 with incomplete data, 8 with advanced osteoarthritis in the contralateral hip, 2 with periprosthetic fractures, 1 with infection, and 1 with dislocation, leaving 116 hips for analysis. Based on the previous literature reporting a normal PO of approximately 2° in healthy adults [11], a PO ≥ 2° was defined as abnormal. Patients were categorized into two groups: U-PO, with affected-side upward tilt of ≥ 2°; and D-PO, with affected-side downward tilt of ≥ 2° (Fig. 1).

**Fig. 2:**
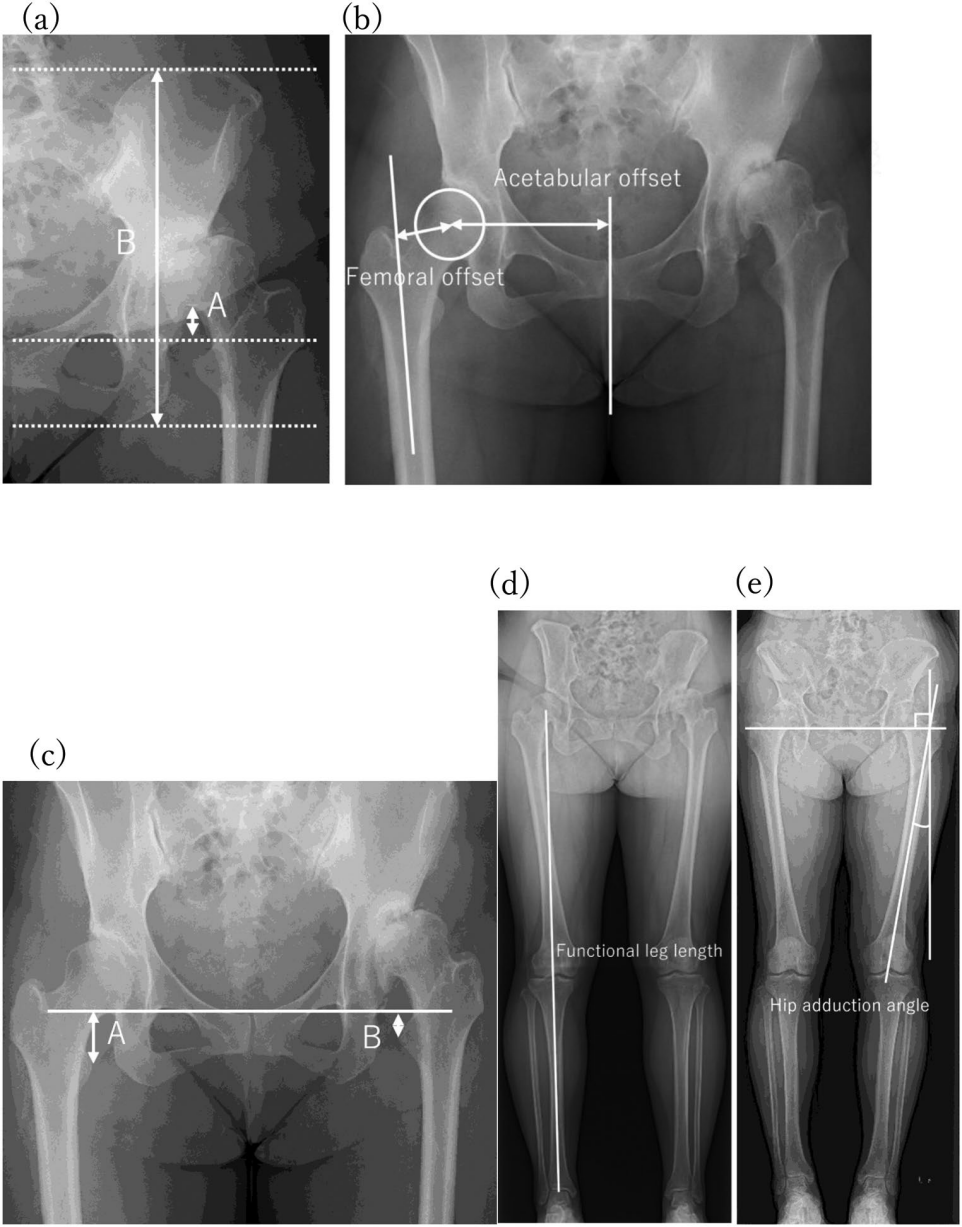
Radiographic evaluation of the lower limbs. (**a**) Clowe index: 5 A/B*100. (**b**) Acetabular offset: **A**, femoral offset: **B**. (**c**) Radiographic leg-length discrepancy: A–B. (**d**) Femoral leg length. (**e**) Hip adduction angle

### Clinical evaluation

Patient demographic data, including age, sex, height, weight, body mass index (BMI), and contralateral hip condition were extracted from medical records. Surgical data, including operative duration, blood loss, cement use, and bone grafting, were also collected. Clinical evaluation included hip Japanese Orthopaedic Association (JOA) score, and joint ROM was measured by a senior orthopedic surgeon using a goniometer.

### Measurement of hip and lower limb radiographic parameters

Preoperative and 1-year postoperative evaluations were performed using anteroposterior hip and standing full-length lower-limb radiographs. Assessment parameters included the Crowe index, acetabular offset, femoral offset, radiographic leg length discrepancy (RLLD), functional leg length discrepancy (FLLD), and hip adduction angle (Fig. 2). The Crowe index indicates the percentage of femoral head subluxation [12]. Acetabular offset was defined as the distance from the perpendicular line through the pubic symphysis to the center of the femoral head. Femoral offset was defined as the distance from the center of the femoral head to the femoral shaft [13]. The RLLD was defined as the difference in the vertical distance between the teardrop of the acetabulum and the lesser trochanter on the operated and non-operated sides [14]. Positive values indicate that the operated side was shorter. Functional leg length was defined as the distance between the center of the femoral head and the center of the ankle joint [9]. The FLLD was obtained by subtracting the value of the affected side from that of the healthy side. The hip adduction angle was defined as the angle between the femoral axis and the vertical line from the acetabular teardrop [7]. Differences between the preoperative and postoperative values were calculated for parameter evaluation.

### Measurement of spinal radiographic parameters

**Fig. 3:**
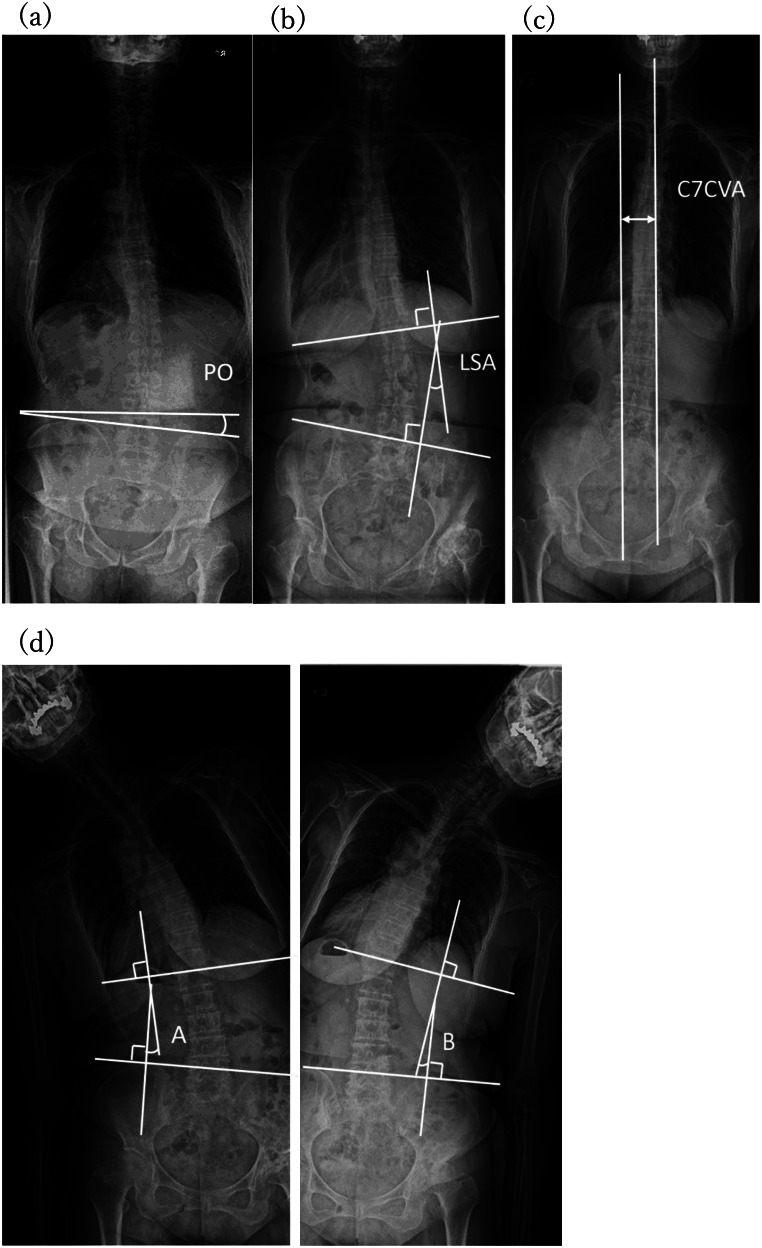
Radiographic evaluation of spinal parameters. (**a**) Pelvic obliquity. (**b**) Lumbar scoliosis angle. (**c**) C7 coronal vertical axis. (**d**) Lumbar bending range: A + B

Evaluations of the spine and pelvis were performed using standing full-spine anteroposterior radiographs captured preoperatively and 1-year postoperatively, focusing on PO, lumbar scoliosis angle, C7 coronal vertical axis (C7CVA), and spinal lateral flexion range of motion. Pelvic obliquity was measured using the method described by Osebold as the angle between the horizontal plane and the line connecting the upper edges of both iliac crests [15]. To assess differences in the horizontal plane, PO was defined as positive for upward inclination in the U-PO group and downward inclination in the D-PO group. Absolute values were not used because they hindered the evaluation of postoperative improvements. The C7CVA and lumbar scoliosis angle were measured using the method described by Nakashima [16]. The C7CVA was defined as the horizontal distance from the sacral centerline to C7, with positive values indicating deviation toward the affected side. The lumbar scoliosis angle was defined as the angle between the extended line from the upper edge of L1 and the line connecting the upper edges of the iliac crests, with positive values indicating convexity toward the affected side (Fig. 3). Similar to the hip and lower limb parameters, postoperative values subtracted from the preoperative values were used for parameter evaluations.

### Surgical technique

Preoperative three-dimensional planning was performed using the ZedHip software (LEXI Co., Tokyo, Japan) to determine the implants aiming to minimize RLLD and offset differences between the operative and nonoperative sides [17]. Implant selection, positioning, and navigation were performed at the discretion of the surgeon. Cement was used for acetabular fixation in cases with unmanageable bone defects and femoral fixation in Dorr type C cases [18]. All procedures used a standard posterior approach, with patients in the lateral decubitus position. The incision was extended proximally from the distal greater trochanter along a gentle curve. The short external rotator and piriformis muscles were incised at the attachment of the greater trochanter and marked for subsequent repair. After the capsule was incised, osteotomy was performed using an oscillator and the femoral head was extracted using a bone head extractor. The femur was retracted and acetabular exposure was achieved using Hohmann retractors. Reaming and cup placement targeted anatomical restoration of the acetabulum. Bone grafting with excised femoral heads was performed if necessary. Femoral stems were chosen for appropriate length, offset, and anteversion, and cement fixation was performed using third-generation techniques. Posterior structures, including the short external rotators and capsules, were repaired through the proximal femoral tunnels. Postoperatively, all patients underwent rehabilitation focusing on gait training, ROM, and strengthening exercises. The femoral implants included 35 Secur-fit Advanced (Stryker, Mahwah, NJ, USA), 30 Exeter (Stryker), 14 Taperloc Microplasty (Zimmer Biomet, Warsaw, IN, USA), 4 Avenir (Zimmer Biomet), 6 CPT (Zimmer Biomet), 3 CMK (Zimmer Biomet), and 24 Initia (Kyocera, Kyoto, Japan) implants. The acetabular components included 43 Trident (Stryker), 17 × 3 RimFit (Stryker), 30 G7 OsseoTi (Zimmer Biomet), and 26 SQRUM (Kyocera) devices. All surgeries were performed by senior surgeons or by junior surgeons under supervision.

### Statistical analysis

Statistical analyses were performed using the Student’s t-test for parametric continuous variables (patient demographic data), Mann–Whitney U test for non-parametric continuous variables (surgical data, hip-spine radiographic parameters), and Fisher’s exact test for categorical variables. Factors affecting residual postoperative pelvic obliquity were evaluated using logistic regression analysis for items with *P* < 0.05. Statistical analysis was performed using the EZR software (Saitama Medical Center, Jichi Medical University, Saitama, Japan). Differences with a *P* < 0.05 were considered to be statistically significant [19].

## Results

The U-PO and D-PO groups comprised 35 and 81 patients, respectively. No significant differences were observed in age, sex, height, weight, BMI, contralateral hip condition, or history of osteotomy between the groups. Regarding the contralateral hip, the osteoarthritis was Tönnis grade 2 or lower and classified as Crowe type 1 [20]. However, the duration of hip disorders was significantly longer in the U-PO group (5.0 [4.0–10.5] years) compared with that in the D-PO group (3.0 [3.0–5.0] years) (*P* = 0.015). There were no significant differences in operative duration, blood loss, cement usage for cups or stems, or bone grafting (Table 1).

**Table 1:** Patient demographics and surgical data

	D-PO group (*n* = 81)	U-PO group (*n* = 35)	*P* value
Demographics data			
Age, yrs (SD)	63.8 (10.4)	65.2 (11.5)	0.502
Sex (%)			0.192
Men	12 (14.8)	9 (25.7)	
Women	69 (85.2)	26 (74.3)	
Height, cm (SD)	154.7 (7.0)	156.3 (9.3)	0.292
Weight, kg (SD)	56.7 (10.8)	60.2 (13.5)	0.152
Body mass index, kg/m2 (SD)	23.5 (3.8)	24.5 (4.2)	0.207
Other side (healthy/ OA/ THA)	56/ 7/ 18	23/ 3/ 8	> 0.99
History of osteotomy (+ / -)	15/66	6/29	> 0.99
Duration of hip disorders, yrs (IQR)	3.0 (3.0–5.0)	5.0 (4.0–10.5)	0.015^※^
Surgical data			
Operative duration, min (IQR)	94 (79–114)	108 (88.5–129.5)	0.057
Blood loss, g (IQR)	362 (231–524)	473 (249.5–607)	0.302
Cup (cement / cementless)	13/68	5/30	> 0.99
Stem (cement / cementless)	26/55	14/21	0.394
Bone implantation (none/ bulk bone/ tip bone)	60/13/8	26/7/2	0.469

D-PO, downward pelvic obliquity; U-PO, upward pelvic obliquity; OA, osteoarthritis; THA, total hip arthroplasty; SD, standard deviation; IQR, interquartile range

On comparing preoperative hip-spine parameters between the groups, the U-PO group exhibited significantly greater acetabular offset (110.5 [103.5–117.0] mm vs. 105.6 [99.3–113.5] mm, *P* = 0.034) and hip adduction angle (9.5° [7.5–10.8°] vs. 5.0° [4.0–7.0°]; *P* < 0.001) than the D-PO group. Compared with the D-PO group, the U-PO group exhibited a significantly longer FLLD on the operative side (4 [0–11] mm vs. -8 [-13–1.5] mm; *P* < 0.001), and a lumbar scoliosis angle with a convexity toward the operative side (2° [0–5.5°] vs. -2° [-6 − 1.6°]; *P* < 0.001) (Table 2).

**Table 2:** Preoperative data between D-PO group and U-PO group

	D-PO group (*n* = 81)	U-PO group (*n* = 35)	*P* value
Crowe index (IQR)	18.6 (0–38.3)	23.1 (8.1–46.6)	0.28
Acetabular offset (operative side), mm (IQR)	105.6 (99.3–113.5)	110.5 (103.5–117.0)	0.034^※^
Femoral offset (operative side), mm (IQR)	34.0 (27.0–40.2)	35.4 (28.5–41.0)	0.798
Hip adduction angle, degree (IQR)	5.0 (4.0–7.0)	9.5 (7.5–10.8)	< 0.001^※^
RLLD, mm (IQR)	14 (5–20)	10 (5–20)	0.657
FLLD, mm (IQR)	4 (0–11)	-8 (-13 – -1.5)	< 0.001^※^
PO, degree (IQR)	3.5 (2.5–4.5)	3.2 (2.0–5.1)	0.535
Lumbar scoliosis angle, degree (IQR)	-2 (-6–1.6)	2 (0–5.5)	< 0.001^※^
C7CVA, mm (IQR)	11 (0–20)	10 (0–19)	0.705
Lumbar bending range, degree (IQR)	12.2 (7.0–17)	10.5 (6.0–17)	0.466

*P<0.05

D-PO, downward pelvic obliquity; U-PO, upward pelvic obliquity; RLLD, radiographic leg length discrepancy; FLLD, functional leg length discrepancy; PO, pelvic obliquity; C7CVA, C7 coronal vertical axis; IQR, interquartile range

When comparing postoperative hip-spine parameters, the significant differences in acetabular offset and lumbar scoliosis angle between the groups disappeared. However, the hip adduction angle was significantly greater in the U-PO group than in the D-PO group (8.0° [6.0–9.0°] vs. 5.5° [4.0–6.0°]; *P* < 0.001), and the FLLD favoring the operative side persisted (1 [-3–8] vs. -2 [-5–0] mm; *P* = 0.002). Pelvic obliquity was also more pronounced in the U-PO group than in the D-PO group (3° [0–4°] vs. 1° [0–3°]; *P* = 0.009) (Table 3).

**Table 3:** Postoperative data between D-PO group and U-PO group

	D-PO group (*n* = 81)	U-PO group (*n* = 35)	*P* value
Acetabular offset (operative side), mm (IQR)	97.5 (94.5–100.7)	97.5 (92.0–103.6)	0.880
Femoral offset (operative side), mm (IQR)	41 (36.5–44.4)	42 (37.9–47.4)	0.443
Hip adduction angle, degree (IQR)	5.5 (4.0–6.0)	8.0 (6.0–9.0)	< 0.001^※^
RLLD, mm (IQR)	0 (0–5)	0 (-2.7–3)	0.189
FLLD, mm (IQR)	1 (-3–8)	-2 (-5–0)	0.002^※^
PO, degree (IQR)	1 (0–3)	3 (0–4)	0.009^※^
Lumbar scoliosis angle, degree (IQR)	0 (-4.9–3.8)	2 (0–4.0)	0.075
C7CVA, mm (IQR)	0 (0–15.6)	0 (0–16.0)	0.818

*P<0.05

D-PO, downward pelvic obliquity; U-PO, upward pelvic obliquity; RLLD, radiographic leg length discrepancy; FLLD, functional leg length discrepancy; PO, pelvic obliquity; C7CVA, C7 coronal vertical axis; IQR, interquartile range

The hip adduction angle decreased significantly in the U-PO group (-1° [-3.3–0.5°] vs. 0° [-1.5–1.5°]; *P* = 0.006). FLLD improved toward a smaller discrepancy in both groups (-4 [-7–3] mm in the D-PO group and 1 [-4–5.5] mm in the U-PO group; *P* = 0.044). The reduction in PO was smaller in the U-PO group (-2.5° [-4.6 – -1.7°] vs. -1.9° [-3.0–1.0°]; *P* = 0.003), and residual PO was more common in the U-PO group than in the D-PO group (60.0% vs. 38.3%; *P* = 0.042) (Table 4).

The total hip JOA hip score was significantly lower in the U-PO group (85 [81–92]) than in the D-PO group (92 [85–96]; *P* = 0.016). The subscores for gait (18 [7, 15–19] vs. 20 [7, 15–19]; *P* = 0.041) and ROM (15 [12.5–18] vs. 18 [7, 15–19]; *P* < 0.001) were also significantly worse in the U-PO group than in the D-PO group (Table 5).

## Discussion

**Fig. 4:**
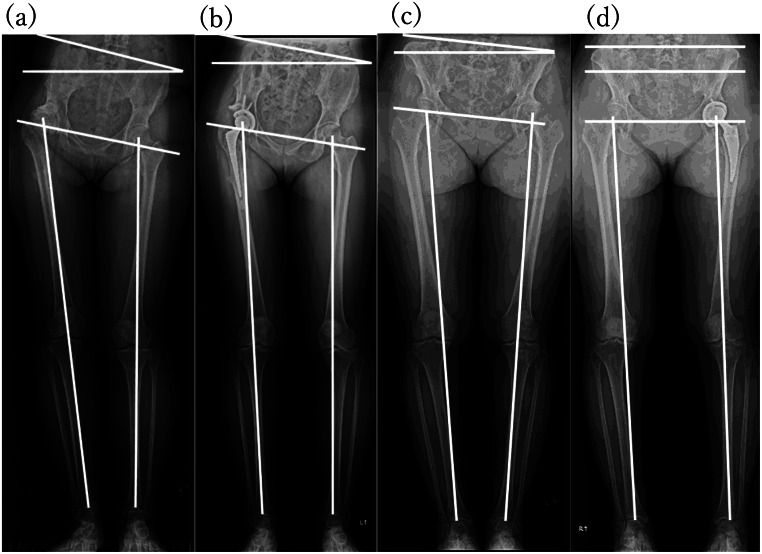
Representative Cases. U-PO Case. A 47-year-old female underwent hybrid total hip arthroplasty (THA) for dysplastic right hip osteoarthritis (DHOA). Preoperative parameters were Crowe index 34.4, pelvic obliquity (PO) 17°, hip adduction angle 12°, radiographic length discrepancy (RLLD) 15 mm, and functional length discrepancy (FLLD) -19 mm (**a**). Postoperative parameters included PO 13°, hip adduction angle 10°, RLLD 0 mm, and FLLD − 17 mm (**b**). Her preoperative hip Japanese Orthopaedic Association (JOA) score was 57 (pain, 20; walking, 15; range of motion, 8; activity of daily living, 14), and the postoperative hip JOA score improved to 83 (pain, 40; gait, 15; range of motion, 14; and activities of daily living, 14). D-PO Case. A 52-year-old female underwent cementless THA for left DHOA. Preoperative parameters were Crowe index 12.1, PO 5°, hip adduction angle 4°, RLLD 10 mm, and FLLD 7 mm (**c**). Postoperative parameters included PO 0°, hip adduction angle 6°, RLLD 0 mm, and FLLD 0 mm (**d**). Her preoperative hip JOA score was 66 (pain, 20; walking, 18; range of motion, 16; activities of daily living, 12), and postoperative hip JOA score significantly improved to 98 (pain, 40; gait, 20; ranges of motion, 20; activities of daily living, 18)

This study investigated the effect of preoperative PO direction on hip function and spine morphology in patients undergoing THA for DHOA. In the U-PO group, THA improved hip adduction angle and FLLD, and the large adduction angle and longer FLLD on the affected side persisted postoperatively. Furthermore, PO improvement in the U-PO group was markedly lower in both rate and magnitude than in the D-PO group, with more residual PO. The postoperative hip function in the U-PO group was inferior, particularly in the gait and ROM categories (Fig. 4).

The cause of D-PO is believed to be compensatory changes due to a shorter limb, with previous studies reporting a greater tilt with a larger LLD [21, 22]. Conversely, U-PO is attributed to morphological issues such as hip adduction contracture [23] and weakened abductor muscles [24]. Regarding the relationship between preoperative PO direction and hip–spine parameters, Ozawa et al. noted that patients with an upward-tilted PO on the affected side showed greater lateral displacement of the femoral head, a subluxated position, larger hip adduction angle, longer FLLD on the affected side, and a lumbar scoliosis angle convex to the affected side. These findings were attributed to adduction contractures due to hip subluxation. Regarding the relationship between leg length and PO, studies have suggested that to maintain coronal balance and upright posture, the spine tends to bend toward the longer leg, which can induce scoliosis on the shorter side. Leg length discrepancies exceeding 9 mm may contribute to lumbar scoliosis [25]. In this study, THA improved RLLD in both groups, eliminating the differences in acetabular offset and resolving lumbar scoliosis convex to the affected side in the D-PO group, thus equalizing the lumbar scoliosis angles between the groups postoperatively. Although the hip adduction angle and FLLD improved in the D-PO group, intergroup differences persisted. These findings suggest that while pelvic parameters improved in patients with upward-tilted PO, the overall lower limb alignment was less effectively corrected. This may indicate a limited impact of THA in such cases.

To our knowledge, no previous studies have focused on how PO changes after THA, specifically considering the preoperative PO direction. Li et al. [8] reported that THA improved PO and LLD in patients with DHOA, with more pronounced effects in cases of severe dislocation, such as Crowe types 3 and 4. Similarly, Abe et al. [6] found improved coronal balance in 89.2% of 195 THA cases, confirming the safety of THA for coronal alignment correction, even with limb elongation. In this study, the D-PO group demonstrated marked improvement in PO, with a higher proportion achieving normalization. The improvement in PO was attributed to the correction of LLD through THA, particularly in the D-PO group in which hip joint mobility was relatively preserved. However, in the U-PO group, the changes in PO and proportion of patients achieving normalization were smaller, indicating that PO was not solely determined by pelvic factors. Although spinal influences were minimal, other contributors such as hip adduction contracture and alignment abnormalities caused by a longer FLLD on the affected side played a role. As it has been reported that hip joints with contracture tend to have more osteophytes [4], it is necessary during surgery to remove osteophytes that may restrict the range of motion, perform sufficient release and resection of the joint capsule and other soft tissues, and, if necessary, release the adductor muscles to address the contracture.

Whether the direction of PO affects hip function after THA has not been clarified. Previous studies have indicated that hip contractures can impede postoperative recovery of ROM and complicate the surgical procedure, potentially affecting implant placement and stability [3]. Moreover, persistent soft tissue contractures have been reported even one year after THA in Crowe type 3 or 4 cases [8]. Research investigating PO, LLD, and hip contractures suggests that LLD first leads to pelvic tilt, subsequently resulting in hip contracture [4]. In this study, the U-PO group exhibited poorer postoperative improvement in hip function, particularly in ROM. This may be due to unresolved preoperative contractures caused by the long duration of hip disorders. Early surgical intervention is crucial, particularly in U-PO cases in which postoperative improvement in hip function is challenging.

An important finding of this study was that the direction of preoperative PO was markedly associated with postoperative hip function and the degree of PO improvement after THA. Previous studies have evaluated radiographic characteristics and hip function based on the direction of preoperative PO, whereas others have assessed the risk factors for residual PO after THA. However, these studies did not clarify the direction and degree of preoperative PO and hip function change after THA. Our study demonstrated that patients with DHOA who exhibited PO with an upward inclination of the affected side preoperatively exhibited poorer improvement in both PO and hip function after THA. These findings address the gap in the literature.

This study has some limitations, including its retrospective, observational, single-institution design, and the potential for data collection bias. However, the surgical technique, perioperative management, and rehabilitation were consistent with little variability. Therefore, it is desirable to eliminate institutional bias through collaborative studies involving other institutions. The follow-up period was short (1 year). A longer follow-up period is needed because the clinical outcomes of patients with PO and residual PO may change over time. The possibility that posture and rotation may have influenced the results cannot be excluded by the evaluation of coronal plane radiographs alone. Kapron et al. [26] found that hip flexion of < 30° had little effect on the axial rotation of the pelvis or sagittal plane. In this study, we positioned the patient in the mid-hip joint position and used an imaging method to reduce interfering factors as much as possible, which we believe is an appropriate evaluation method. Radiographic and functional LLDs were investigated; however, subjective LLD was not assessed. Although we were able to evaluate hip function, assessing patient satisfaction remains a task for future studies.

## Conclusion

This study evaluated the impact of the preoperative PO direction on hip function and spinal and lower limb parameters in patients with DHOA who underwent THA. In patients with U-PO, marked postoperative hip adduction angles and FLLDs persisted. Additionally, postoperative hip function, particularly ADL and ROM, was poor, and PO improvement was limited. These findings highlight the importance of early interventions to prevent U-PO progression.

**Table 4:** Comparison of variations between D-PO group and U-PO group

	D-PO group (*n* = 81)	U-PO group (*n* = 35)	*P* value
ΔAcetabular offset (operative side), mm (IQR)	-7.2 (-16.0 – -0.5)	-11.0 (-19.3 – -7.5)	0.068
ΔFemoral offset (operative side), mm (IQR)	8.4 (1.6–14.0)	7.0 (2.5–12.5)	0.950
ΔHip adduction angle, degree (IQR)	0 (-1.5–1.5)	-1 (-3.3–0.5)	0.006^※^
ΔRLLD, mm (IQR)	-12 (-17 – -5)	-10 (-19 – -5)	0.847
ΔFLLD, mm (IQR)	-4 (-7–3)	1 (-4–5.5)	0.044^※^
PO (improve/ residual)	50/31	14/21	0.042^※^
ΔPO, degree (IQR)	-2.5 (-4.6 – -1.7)	-1.9 (-3.0–1.0)	0.003^※^
ΔLumbar scoliosis angle, degree (IQR)	-2.0 (-5.5–0.6)	0.6 (-0.6–3.0)	0.081
ΔC7CVA, mm (IQR)	0 (-12–1)	0 (-10–0)	0.842

*P<0.05

D-PO, downward pelvic obliquity; U-PO, upward pelvic obliquity; RLLD, radiographic leg length discrepancy; FLLD, functional leg length discrepancy; PO, pelvic obliquity; C7CVA, C7 coronal vertical axis; IQR, interquartile range

**Table 5:** Hip JOA score between D-PO group and U-PO group

	D-PO group (*n* = 81)	U-PO group (*n* = 35)	*P* value
preoperative			
Pain (IQR)	20 (20–20)	20 (2–30)	0.396
Gait (IQR)	15 (10–18)	15 (10–15)	0.134
Range of motion (IQR)	13 (10–17)	11 (8–13.5)	0.015^※^
Activities of Daily Living (IQR)	14 (10–16)	12 (11–14)	0.389
Total (IQR)	61 (52–69)	57 (51–66)	0.294
postoperative			
Pain (IQR)	40 (40–40)	40 (40–40)	0.929
Gait (IQR)	20 (15–20)	18 (15–20)	0.041^※^
Range of motion (IQR)	18 (15–20)	15 (12.5–18)	< 0.001^※^
Activities of Daily Living (IQR)	18 (16–20)	16 (14–18)	0.171
Total (IQR)	92 (85–96)	85 (81–92)	0.016^※^

*P<0.05

JOA, Japanese Orthopaedic Association; D-PO, downward pelvic obliquity; U-PO, upward pelvic obliquity; IQR, interquartile range

## Data Availability

No datasets were generated or analysed during the current study.

## Abbreviations

PO: Pelvic obliquity
DHOA: Dysplastic hip osteoarthritis
THA: Total hip arthroplasty
U-PO: Upward pelvic obliquity
D-PO: Downward pelvic obliquity
LLD: Leg length discrepancy
ROM: Range of motion
RLLD: Radiographic leg length discrepancy
FLLD: Functional leg length discrepancy
C7CVA: C7 coronal vertical axis
